# Cardiovascular disease biomarkers are associated with declining renal function in type 2 diabetes

**DOI:** 10.1007/s00125-017-4297-0

**Published:** 2017-05-20

**Authors:** Sara J. Jenks, Bryan R. Conway, Stela McLachlan, Wei Leng Teoh, Rachel M. Williamson, David J. Webb, Paul Welsh, Naveed Sattar, Mark W. J. Strachan, Jackie F. Price

**Affiliations:** 1 Department of Clinical Biochemistry, Royal Infirmary of Edinburgh, Edinburgh, UK; 2 Centre for Population Health Sciences, University of Edinburgh, Old Medical School, Teviot Place, Edinburgh, EH8 9AG UK; 3 University of Edinburgh/British Heart Foundation Centre for Cardiovascular Science, Queens Medical Research Institute, University of Edinburgh, Edinburgh, UK; 4 Metabolic Unit, Western General Hospital, Edinburgh, UK; 5 Institute of Cardiovascular and Medical Sciences, University of Glasgow, Glasgow, UK

**Keywords:** Cardiovascular disease, Chronic kidney disease, Diabetic nephropathy, Troponin, Type 2 diabetes mellitus

## Abstract

**Aims/hypothesis:**

We investigated whether biochemical cardiovascular risk factors and/or markers of subclinical cardiovascular disease were associated with the development of reduced renal function in people with type 2 diabetes.

**Methods:**

A cohort of 1066 Scottish men and women aged 60–74 years with type 2 diabetes from the Edinburgh Type 2 Diabetes Study were followed up for a median of 6.7 years. New-onset reduced renal function was defined as two eGFRs <60 ml^−1^ min^−1^ (1.73 m)^−2^ at least 3 months apart with a > 25% decline from baseline eGFR. Ankle brachial pressure index (ABI), N-terminal pro-B-type natriuretic peptide (NT-proBNP) and high-sensitivity troponin T (hsTnT) were measured at baseline. Pulse wave velocity (PWV) and carotid intima media thickness were measured 1 year into follow-up. Data were analysed using Cox proportional hazards models.

**Results:**

A total of 119 participants developed reduced renal function during follow-up. ABI, PWV, NT-proBNP and hsTnT were all associated with onset of decline in renal function following adjustment for age and sex. These associations were attenuated after adjustment for additional diabetes renal disease risk factors (systolic BP, baseline eGFR, albumin:creatinine ratio and smoking pack-years), with the exception of hsTnT which remained independently associated (HR 1.51 [95% CI 1.22, 1.87]). Inclusion of hsTnT in a predictive model improved the continuous net reclassification index by 0.165 (0.008, 0.286).

**Conclusions/interpretation:**

Our findings demonstrate an association between hsTnT, a marker of subclinical cardiac ischaemia, and subsequent renal function decline. Further research is required to establish the predictive value of hsTnT and response to intervention.

## Introduction

The development of reduced renal function and chronic kidney disease (CKD) in people with type 2 diabetes predicts an increase in both morbidity and mortality rates [1]. As a result, improved methods for identifying high-risk individuals need to be established to enable early, targeted interventions to prevent decline of renal function and improve treatment outcomes. Early research investigating the development of CKD in people with type 2 diabetes indicated that the predominant aetiology was diabetic nephropathy [2], the hallmark of which is the development of albuminuria. Albumin:creatinine ratio (ACR) is, therefore, commonly used clinically to identify individuals with type 2 diabetes who have developed CKD and are at high risk of progression [2, 3]. However, more recently, a growing number of studies have demonstrated that many people with type 2 diabetes develop a progressive deterioration of renal function in the absence of albuminuria [4, 5].

It has been proposed that many people with type 2 diabetes develop impaired renal function as a result of vascular disease, rather than classical diabetic nephropathy, consistent with ACR being a poor kidney disease biomarker in many individuals [5]. If correct, then it would be reasonable to hypothesise that cardiovascular disease (CVD) biomarkers may improve on risk stratification using conventional risk factors for diabetic CKD. Support for this hypothesis comes from previous studies demonstrating an association between the development of end-stage renal disease and biochemical markers of CVD, N-terminal pro-B-type natriuretic peptide (NT-proBNP) and high-sensitivity troponin T (hsTnT), in people with type 2 diabetes, even after adjustment for eGFR and albuminuria [6, 7]. Markers of subclinical atherosclerosis have also been linked with renal disease progression including ankle brachial pressure index (ABI) and carotid intima media thickness (cIMT), along with markers of arterial stiffness, such as pulse wave velocity (PWV). However, some of the evidence is conflicting and there have been no large longitudinal studies focusing on people with type 2 diabetes [8–12].

Our study set out to investigate the association between CVD biomarkers (blood-based and non-blood-based) and the onset of impaired renal function in a prospective cohort of people with type 2 diabetes (the Edinburgh Type 2 Diabetes Study [ET2DS]). We hypothesised that increased levels of individual CVD risk factors and/or markers of clinical or subclinical CVD would be associated with the development of clinically significant decline in renal function and, further, that some of these markers would add predictive value beyond recognised risk factors for renal disease.

## Methods

We performed a prospective cohort study using participants of the ET2DS. Recruitment and baseline examination for the ET2DS has been described previously [13, 14]. Briefly, the ET2DS recruited 1066 men and women aged 60–74 years with established type 2 diabetes living in the Lothian region of Scotland between 2006 and 2007. Participants were randomly selected by sex and 5 year age bands from the Lothian diabetes register which is a comprehensive database of people with type 2 diabetes living in Lothian. The database includes individuals attending both hospital diabetes clinics and those managed solely in primary care. Of the 5454 individuals who were invited to participate, 1066 were recruited to the study and attended the baseline research clinic. The ET2DS population has been shown previously to be largely representative, in terms of age, HbA_1c_, duration of diabetes and insulin treatment, of all those randomly selected to participate [15]. Approval for ET2DS has been given by the Lothian Medical Research Ethics Committee and all participants have given written consent, including for follow-up examinations and review of medical records.

At the baseline ET2DS clinic attendance, demographic, medical history and clinical variables were measured including BP and ABI. Following an overnight fast, venous blood and urine samples were obtained for biochemical analysis. The presence of CVD was determined using a combination of self-report, record linkage and ECG data. Presence of CVD was indicated by a baseline history of transient ischaemic attack (TIA), stroke, myocardial infarction (MI), angina and/or intermittent claudication, defined according to previously reported criteria [15].

All blood samples were separated and frozen at −80°C within 1 h of venepuncture, except for samples for creatinine, HbA_1c_ and glucose analyses for which fresh samples were used. ACR was measured on a fresh urine sample. All samples were analysed according to standard protocols in the Department of Biochemistry, Western General Hospital, Edinburgh, UK.

Plasma C reactive protein (CRP), NT-proBNP and hsTnT concentrations were measured at the University of Glasgow laboratories. CRP was assayed using a high-sensitivity immunonephelometric assay with a BN ProSpec nephelometer (Dade Behring, Milton Keynes, UK) [16]. NT-proBNP and hsTnT were measured using the Elecsys 2010 electrochemiluminescence methods (Roche Diagnostics, Burgess Hill, UK) calibrated using the manufacturer’s reagents. Plasma samples for NT-proBNP and hsTnT had been stored at −80°C for up to 4 years prior to analysis. Manufacturer’s controls were used with limits of acceptability defined by the manufacturer. The between batch CVs for NT-proBNP were 6.7% at a concentration of 131 ng/l and 4.9% at 4602 ng/l. For hsTnT the between batch CVs were 3.7% at 25.2 ng/l and 2.2% at 2254 ng/l.

For measurement of ABI, brachial, posterior tibial and dorsalis pedis systolic BP (SBP) were measured in both arms and both feet using either an aneroid, 6-in. dial, desk standing sphygmomanometer or a mercury sphygmomanometer (WBIC, Wenzhou, China). ABI was calculated using the lowest ankle pressure as the numerator and the highest brachial pressure as the denominator. ABI results of >1.3 (*n* = 26) were excluded, as very high values may indicate arterial stiffness in diabetic populations [17].

Participants were assessed for the presence of diabetic retinopathy (DR) using retinal photography. Pupillary dilatation was carried out followed by standard seven-field non-stereoscopic colour photographs being taken of both eyes at 35° using a high-resolution digital retinal camera. Two trained optometrists graded all the photographs, working independently and according to the scale described by the Early Treatment Diabetic Retinopathy Study research group [18]. Inter- and intraobserver variations and the validity of this grading system have previously been evaluated [19]. For each eye, the maximum grade in any of the seven photographic fields was determined for each of the characteristic lesions of DR and was used in defining the final retinopathy levels. The retinopathy level for a participant was assigned based on the severity scores of the worst eye. Any discrepancies between the scores assigned by the two graders were resolved through discussion between the graders with any unresolved discrepancies being reviewed and arbitrated by an ophthalmologist.

One year after recruitment, all participants who were still living were invited to attend a further research clinic for assessment of PWV and cIMT. A total of 939 participants attended. Of the 133 participants not attending, 15 had died and the remainder were uncontactable, or unable or unwilling to attend. The baseline characteristics of participants attending the year 1 clinic were similar to those of the total ET2DS population suggesting that this group remained largely representative of the target general population with type 2 diabetes [13].

PWV measurements were carried out after participants had been supine for at least 25 min by obtaining the arterial pulse wave form at the common carotid and femoral arteries using the Sphygmo Cor system version 8.0 (At Cor Medical Pty, Sydney, NSW, Australia). The time delay between the arrival of the pulse wave at the two points was measured. A tape measure was used to determine the distance between the carotid and sternal notch and the distance between sternal notch and the femoral site. ‘Direct path length’ was calculated by subtracting the carotid–sternal notch distance from sternal notch–femoral distance. Where possible, all measurements were made in duplicate and mean values calculated [20].

cIMT, defined as the distance between the lumen–intima interface and the media–adventitia interface, was measured bilaterally in three separate images of the common carotid artery using a Sonoline Elegra Ultrasound Imaging System (Siemens Medical Systems, Issaquah, WA, USA). Mean cIMT was calculated for the right and left common carotid arteries and the higher of these two values was used for analyses [21].

### Follow-up

The Lothian laboratory and Lothian diabetes register databases were interrogated and all outpatient or primary care serum creatinine results from 2005 to May 2014 were identified. eGFR was calculated from serum isotope dilution MS–traceable creatinine results using the Chronic Kidney Disease Epidemiology Collaboration (CKD-EPI) equation [22].

New-onset of reduced renal function was defined as two eGFRs <60 ml^−1^ min^−1^ (1.73 m)^−2^ at least 3 months apart with a > 25% decline in eGFR from baseline as per the Kidney Disease Improving Global Outcomes (KDIGO) clinical practice guideline for the evaluation and management of CKD [23].

### Data analysis

Results are expressed as means (± SD) unless otherwise stated. Any data that did not conform to the normal distribution were log_10_-transformed (e.g. NT-proBNP, hsTnT, ACR, smoking pack-years) prior to further analysis. Comparison of variables between groups was performed using ANOVA or χ^2^ test for continuous and categorical values, respectively. The association between baseline variables and new-onset renal function decline was assessed by Cox proportional hazards. HRs for continuous variables relate to a 1 SD increase. Model fit was assessed using C statistics, continuous net reclassification index (NRI) and integrated discrimination index (IDI). Continuous NRI and IDI were calculated as it has been suggested that changes in C statistic are not sensitive enough to accurately reflect the added value that new biomarkers bring to a model [24]. Continuous NRI provides an indication of how many individuals are ‘reclassified’ in the correct direction by the new model compared with the reference model; whilst IDI quantifies the difference in discrimination slopes between the reference model and new model.

All analyses were performed using the SPSS statistics software package (version 21.0; IBM, Armonk, NY, USA) apart from C statistic, continuous NRI and IDI calculations which were performed using R (version 3.1.1, R Foundation for Statistical Computing, Vienna, Austria). A *p* value of <0.05 was considered to be significant.

## Results

Baseline and follow-up renal function data were available for 1048 of the 1066 ET2DS participants. Participants with evidence of CKD at baseline (*n* = 347, 33%), defined as either an eGFR <60 ml^−1^ min^−1^ (1.73 m)^−2^ on two or more consecutive eGFRs at least 3 months apart and/or albuminuria (ACR >2.5 mg/mmol in men; >3.5 mg/mmol in women) on two out of three consecutive urine samples, were excluded from further analysis. The median participant follow-up was 6.7 years with an interquartile range of 6.4–7.1 years.

Of the 701 participants who did not have evidence of CKD at baseline, 119 (17%) developed reduced renal function during follow-up. Serial ACR results for these 119 participants were specifically reviewed and, of these, 101 (85%) did not have preceding albuminuria.

A comparison of baseline variables in participants according to whether or not they developed reduced renal function is shown in Table 1. As expected, participants who were older, had a lower baseline eGFR, longer diabetes duration and higher HbA_1c_ were more likely to develop renal impairment. There was no association with baseline ACR. Of the cardiovascular biomarkers analysed, the presence of CVD, lower ABI, higher PWV, NT-proBNP and hsTnT were all associated with the onset of reduced renal function.

**Table 1:** Comparison of baseline variables in participants according to onset of renal function decline

	Renal function decline			
	*N*	Yes (*n* = 119)	No (*n* = 582)	*p*
Established kidney disease risk factors				
Sex (men), *n* (%)	701	51 (43%)	302 (52%)	0.059
Age, years	701	68.8 ± 3.7	67.0 ± 4.1	<0.001
SBP, mmHg	701	135 ± 17	132 ± 15	0.064
eGFR, ml^−1^ min^−1^ (1.73 m)^−2^	701	80.2 ± 9.5	87.2 ± 10.4	<0.001
Diabetes duration^a^, years	694	6.9 ± 1.0	5.3 ± 1.0	0.002
ACR, *n* (%)	701			
≤ 0.5 mg/mmol		81 (68%)	422 (73%)	
0.6–1.5 mg/mmol		24 (20%)	106 (18%)	0.159
1.6–2.5 mg/mmol		11 (9%)	38 (7%)	
2.6–3.5 mg/mmol		4 (3%)	15 (3%)	
HbA_1c_ (DCCT%)	701	7.6 ± 1.2	7.3 ± 1.1	0.003
HbA_1c_ (IFCC mmol/mol)		60 ± 9.5	56 ± 9.3	
Smoking, *n* (%)	701			
Never		47 (39%)	239 (41%)	
Former		55 (46%)	264 (45%)	0.607
Current		18 (15%)	78 (13%)	
Medications, *n* (%)	699			
ACE/ARB		83 (70%)	347 (60%)	0.043
Antiplatelets	699	73 (61%)	372 (64%)	0.564
Statins	699	99 (83%)	468 (81%)	0.525
Prevalent microvascular disease	685			
Retinopathy, *n* (%)				
None		78 (66%)	412 (73%)	
Mild		37 (31%)	142 (25%)	0.256
Moderate/severe		4 (3%)	12 (2%)	
Prevalent CVD				
CVD, *n* (%)	701	47 (40%)	163 (28%)	0.016
CVD risk factors				
BMI, kg/m^2^	701	30.9 ± 5.5	31.3 ± 5.3	0.400
DBP, mmHg	701	69 ± 9	70 ± 9	0.384
Total cholesterol, mmol/l	690	4.3 ± 1.0	4.3 ± 0.9	0.727
HDL-cholesterol, mmol/l	690	1.3 ± 0.3	1.3 ± 0.4	0.739
Cholesterol:HDL-cholesterol ratio	689	3.4 ± 1.0	3.5 ± 1.1	0.686
CRP^a^, mg/l	683	2.2 ± 3.4	1.7 ± 3.2	0.069
Subclinical markers of CVD				
cIMT (year 1), mm	613	1.00 ± 0.10	0.98 ± 0.17	0.572
PWV (year 1), mm/s	583	9.8 ± 2.3	9.2 ± 2.1	0.013
ABI	681	0.96 ± 0.18	1.00 ± 0.15	0.013
Biochemical markers of CVD				
NT-proBNP^a^, ng/l	688	82.9 ± 2.8	58.7 ± 2.9	0.001
hsTnT^a^, ng/l	695	9.8 ± 1.7	8.4 ± 1.6	0.001

Data presented are mean ± SD for continuous variables

^a^Geometric mean ± SD

ANOVA for continuous variables; *χ* ^2^ for categorical variables

ARB, angiotensin-receptor blocker

The Cox regression analysis of the relationship between baseline variables and the onset of reduced renal function is shown in Table 2. There was a log-linear association between all the CVD biomarkers and reduced renal function with no evidence of non-linearity. Of the cardiovascular biomarkers, higher NT-proBNP, hsTnT and PWV, and lower ABI were all associated with renal function decline after adjustment for age and sex (Model 1)*.* Further adjustment for all the classical diabetic kidney disease risk factors (HbA_1c_, SBP, diabetes duration, ACR, eGFR, smoking pack-years) was carried out (Model 2). Following this adjustment, higher hsTnT (HR 1.51 [95% CI 1.22, 1.87]; *p* < 0.001) continued to be strongly associated with the onset of renal impairment. The relationship between the other CVD biomarkers and renal function decline was attenuated to borderline. The main loss of significance for NT-proBNP occurred following the introduction of baseline eGFR into the model; whilst it was the introduction of smoking pack-years into the model that attenuated the relationship between PWV and ABI with decline in renal function (data not shown). There was no evidence of any interaction effects for any of the main adjustment variables (data not shown).

**Table 2:** Cox regression analysis showing the relationship between cardiovascular and other risk factors and the development of renal function decline

	Model 1			Model 2		
	HR	95% CI	*p*	HR	95% CI	*p*
Established kidney disease risk factors						
Age	1.58	1.32, 1.90	<0.001	1.39	1.14, 1.70	0.001
Sex (women)	1.55	1.08, 2.23	0.018	2.01	1.35, 2.99	0.001
SBP	1.15	0.96, 1.37	0.129	1.24	1.03, 1.49	0.021
HbA_1c_	1.26	1.10, 1.45	0.001	1.30	1.09, 1.54	0.003
log_10_ Diabetes duration	1.38	1.15, 1.66	0.001	1.32	1.07, 1.62	0.009
eGFR	0.50	0.42, 0.60	<0.001	0.47	0.39, 0.57	<0.001
log_10_ ACR +1	1.14	0.96, 1.34	0.127	1.07	0.87, 1.25	0.662
log_10_ Smoking pack-years +1	1.23	1.01, 1.49	0.038	1.33	1.09, 1.64	0.006
Prevalent microvascular disease						
Retinopathy						
No retinopathy						
Mild	1.32	0.89, 1.95	0.168	0.96	0.64, 1.44	0.836
Moderate/severe	1.60	0.58, 4.40	0.359	0.78	0.26, 2.25	0.630
Prevalent CVD						
CVD (history of MI, angina, claudication, TIA/stroke)	1.56	1.09, 2.30	0.017	1.20	0.79, 1.80	0.394
CVD risk factors						
DBP	1.02	0.84, 1.23	0.883	0.96	0.77, 1.20	0.722
Total cholesterol	0.95	0.79, 1.15	0.615	0.90	0.74, 1.09	0.261
HDL-cholesterol	0.90	0.74, 1.09	0.294	0.89	0.73, 1.09	0.263
Cholesterol:HDL-cholesterol ratio	1.02	0.85, 1.22	0.863	0.99	0.81, 1.22	0.936
log_10_ hsCRP	1.19	0.99, 1.43	0.070	1.35	0.92, 1.99	0.130
Subclinical markers of CVD						
cIMT	1.06	0.87, 1.29	0.581	1.07	0.88, 1.30	0.492
PWV	1.25	1.03, 1.53	0.026	1.09	0.99, 1.19	0.077
ABI	0.83	0.70, 0.97	0.022	0.48	0.14, 1.66	0.247
Biochemical markers of CVD						
log_10_ NT-proBNP	1.35	1.12, 1.62	0.002	1.18	0.96, 1.45	0.108
log_10_ hsTnT	1.56	1.30, 1.87	<0.001	1.51	1.22, 1.87	<0.001

Data are HR (95% CI) by Cox regression analysis

HR for continuous variables relate to a 1 SD increase

Model 1, adjusted for age and sex; Model 2, adjusted for established CKD risk factors (age, sex, SBP, HbA_1c_, log_10_ ACR+1, baseline eGFR, duration of diabetes, log_10_ smoking pack-years +1)

We did not find evidence of an association between the baseline presence of DR, lipid levels, diastolic BP (DBP), CRP and cIMT with decline in renal function.

**Fig. 1:**
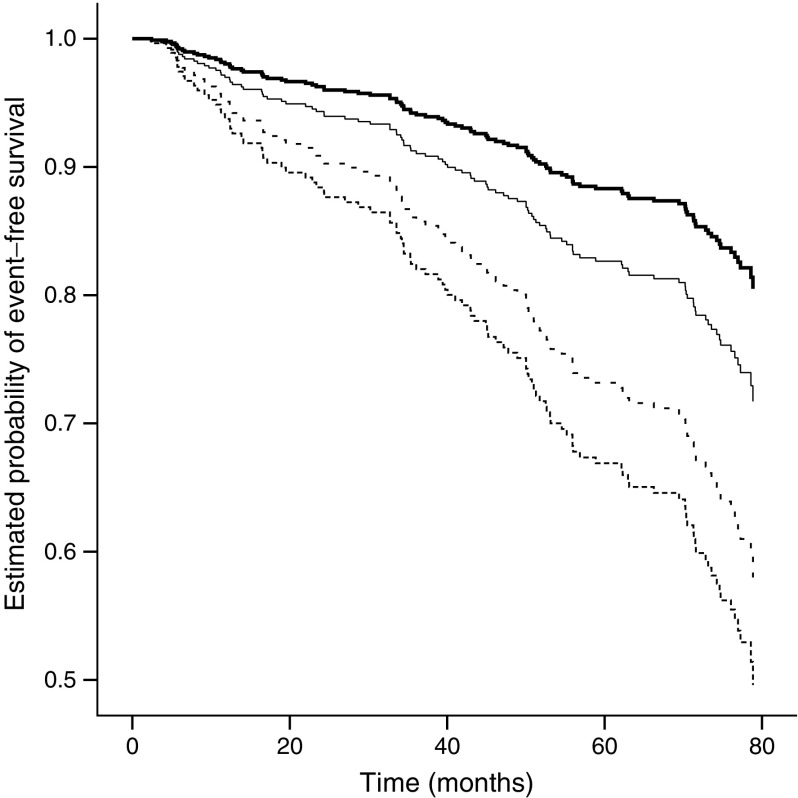
Kaplan–Meier curve demonstrating onset of renal function decline stratified according to baseline hsTnT quartile (for men Q1 3.28–8.19, Q2 8.20–10.24, Q3 10.25–13.60, Q4 13.61–284.30 ng/l; for women Q1 2.90–5.31, Q2 5.32–6.87, Q3 6.88–9.13, Q4 9.14–39.94 ng/l). Thick black line, Q1; thin black line, Q2; dashed line, Q3; dotted line, Q4

Figure 1 displays the onset of renal function decline over time stratified according to baseline hsTnT quartiles. Sex specific quartiles were used to correct for the lower hsTnT levels found in women [25]. The relationship between hsTnT quartile and the onset of decline in renal function was consistent across both sexes and therefore a single Kaplan–Meier plot with the quartiles combined for both sexes is shown. The HR for participants in the highest baseline quarter was 1.60 (95% CI 1.03, 2.50; *p* = 0.038) for women and 2.93 (1.43, 6.03; *p* = 0.003) for men.

The incremental benefit of adding hsTnT to a baseline model of established kidney disease risk factors (age, sex, SBP, HbA_1c_, diabetes duration, baseline ACR, eGFR and smoking pack-years) was assessed. Following the addition of hsTnT to the model there was a non-significant improvement in C statistic from 0.772 (95% CI 0.717, 0.829) to 0.783 (0.728, 0.837). There was a significant improvement in continuous NRI of 0.165 (0.008, 0.286; *p* = 0.04) and IDI of 0.029 (0.002, 0.071; *p* = 0.007).

## Discussion

To our knowledge this is the first study to examine such a comprehensive range of CVD biomarkers (both physical and blood-based cardiovascular phenotyping) and their relationship with the onset of renal function decline in a large longitudinal study of people with type 2 diabetes.

Our study confirms the tremendous burden of kidney disease for people with type 2 diabetes with 347 (33%) of the 1066 participants in the ET2DS cohort having CKD at baseline and a further 119 (17%) developing a clinically relevant decline in renal function during follow-up.

It has traditionally been believed that significant albuminuria occurs prior to the development of functional renal impairment in CKD in people with type 2 diabetes. Therefore, ACR is used to screen for the development of CKD and identify individuals at high risk of progression [26, 27]. However, of the 119 participants who developed reduced renal function during follow-up, the largest proportion (*n* = 101, 85%) did not develop albuminuria prior to a decline in renal function during the follow-up period. The proportion is higher than that found in the United Kingdom Prospective Diabetes Study (UKPDS) where 51% of those developing renal impairment did not have preceding albuminuria [28]. This may be due to the fact that participants with albuminuria at baseline were excluded, the participants in our cohort were older or that more people are now diagnosed with diabetes earlier so that progression of renal disease has been slowed [29]. Furthermore, almost two-thirds of participants were prescribed renin-angiotensin system inhibitors at baseline and this may have masked the presence of albuminuria. Finally, we do not have information regarding changes in medication during the study, it is possible that for some participants a decline in eGFR may reflect the introduction of renin-angiotensin system inhibitor or diuretic rather than progression of intrinsic renal disease.

The presence of microvascular diabetes complications, such as DR, has also traditionally been considered to be a risk factor for the development of CKD in people with type 2 diabetes [30]. However, our study did not find any evidence of an association between the baseline presence of DR and decline in renal function. Overall, the large number of participants developing renal impairment in the absence of either albuminuria or retinopathy indicates that the traditional clinical paradigm of diabetic nephropathy needs to be re-evaluated as it does not appear to reflect the pathological process occurring in the majority of people with type 2 diabetes and declining renal function [5].

### Association between renal function decline and cardiovascular biomarkers/risk factors

Higher levels of hsTnT, NT-proBNP and PWV, and lower ABI levels were all associated with renal function decline following adjustment for age and sex. After further adjustment for classical kidney disease risk factors, including eGFR and smoking pack-years, the strong association between baseline hsTnT level and renal function decline persisted, with men in the highest hsTnT quartile demonstrating an almost threefold increase in risk. Importantly, the addition of hsTnT to a risk prediction model also provided incremental value over standard CKD risk factors in predicting renal function decline with a substantial continuous NRI improvement of 16.3% (*p* = 0.02). These results indicate that hsTnT may be useful for identifying individuals at increased risk of developing a clinically significant decline in renal function. In addition, hsTnT may have utility in clinical trials through improving the identification of participants at high risk of developing renal disease and, as a result, enabling more effective evaluation of interventions.

There are few prior data on the prognostic value of cardiovascular biomarkers in people with type 2 diabetes for the prediction of decline in renal function. Support for our findings comes from studies demonstrating an association between troponin levels and diabetic nephropathy [7] and progression to end-stage renal disease [6].

In addition to providing quantitative data on risk prediction, our findings that subclinical changes in hsTnT precede the development of renal impairment in people with type 2 diabetes also raise questions about the pathophysiological mechanisms underlying the development of CKD.

The physiological basis for an association between hsTnT and incident CKD is not well established. hsTnT is to some extent cleared by the kidneys and hsTnT levels must therefore be interpreted in relation to renal function [31, 32]. However, the relationship between hsTnT and renal outcomes remained strong (*p* < 0.001) even after adjusting for baseline eGFR. hsTnT is a marker of myocardial injury and micronecrosis, it is therefore possible that cardiac vascular disease, reflected by slightly raised hsTnT levels, could be paralleled by vascular disease in other organs, including the kidneys [33].

Baseline levels of PWV and ABI were associated with the onset of renal impairment but these associations were attenuated to the null on multivariate analysis, with the main loss of significance occurring following adjustment for smoking pack-years. There was no evidence of any association between cIMT and renal impairment. Our findings are in contrast to prospective studies on the general population which have found ABI, PWV and cIMT to be independently associated with renal function decline and/or the onset of CKD [34–36]. The differing results could be due to the careful adjustment for smoking history based on calculated pack-years in our study or the different course/drivers of renal disease in the general population relative to people with diabetes. Furthermore, cIMT and PWV were measured 1 year into follow-up in our study, which may have limited their predictive potential. Finally, our cohort is modest in size and event numbers, and therefore not sufficiently powered to detect a small association between these cardiovascular biomarkers and renal disease.

### Strengths and limitations

This study has several strengths including the prospective nature, length of follow-up and large cohort. Furthermore, the review of laboratory records to obtain serial serum creatinine results ensured that the follow-up data were almost entirely complete and that a rigorous, internationally recognised, definition of decline in renal function based on more than one eGFR result could be applied [23]. Additionally, the majority of the participants had undergone detailed cardiovascular phenotyping, allowing us to investigate both structural and biochemical cardiovascular biomarkers simultaneously for the first time.

We acknowledge that our study has limitations. First, we used serum creatinine to calculate eGFR rather than obtaining a directly measured GFR. eGFRs may be less accurate in people with higher levels of renal function; therefore, we employed the CKD-EPI equation, which has been shown to be more accurate at estimating GFR at higher levels of renal function than the widely used Modification of Diet in Renal Disease equation [22]. Furthermore, to avoid inclusion of participants who crossed the eGFR <60 ml^−1^ min^−1^ (1.73 m)^−2^ threshold despite only a small absolute reduction in renal function, we only included those who also had >25% reduction from baseline renal function as recommended by KDIGO [23]. Second, the PWV and cIMT measurements were performed 1 year into follow-up and it is therefore possible that this may have impacted on the ability of our study to assess the association between these variables and renal disease. However, exclusion of the 18 participants who developed an endpoint less than 1 year into follow-up did not significantly alter the results of the analysis for either cIMT or PWV (data not shown). Third, because of limited baseline plasma availability it was not possible to carry out NT-proBNP and hsTnT measurements on all participants. Fourth, plasma samples were stored frozen prior to NT-proBNP and troponin analysis. It has previously been reported that both NT-proBNP and troponin T levels may fall slightly during long-term (>12 months) storage; therefore, it is possible that some sample degradation may have occurred. However, studies have shown that levels after long-term storage remain highly correlated with baseline levels and that the amount of degradation is likely to be small (<6%) [37, 38]. Finally, due to the observational design of this study, it was not possible to evaluate the potential for causality in any of the reported associations.

In conclusion, this study demonstrates the significant burden of CKD for people with type 2 diabetes and the need for new, more efficacious biomarkers to enable improved identification of individuals at high risk of kidney disease development and progression. The findings in this study of an association between subclinical CVD biomarkers/risk factors, such as hsTnT, and subsequent decline in renal function is therefore important. Further research is required to establish the predictive value and thus clinical utility of these biomarkers (and notably automated hsTnT measurements are widely available in most laboratories) and their response to interventions known to lessen risk of CKD in people with diabetes.

## Abbreviations

ABI: Ankle brachial pressure index
ACR: Albumin: creatinine ratio
cIMT: Carotid intima media thickness
CKD: Chronic kidney disease
CRP: C reactive protein
CVD: Cardiovascular disease
DBP: Diastolic BP
DR: Diabetic retinopathy
ET2DS: Edinburgh Type 2 Diabetes Study
hsTnT: High-sensitivity troponin T
IDI: Integrated discrimination index
MI: Myocardial infarction
NRI: Net reclassification index
NT-proBNP: N-terminal pro-B-type natriuretic peptide
PWV: Pulse wave velocity
SBP: Systolic BP
TIA: Transient ischaemic attack
